# Antifungal Efficacy of 3D-Cultured Palatal Mesenchymal Stem Cells and Their Secreted Factors against *Candida albicans*

**DOI:** 10.1021/acsinfecdis.5c00657

**Published:** 2025-09-19

**Authors:** Mesude Bicer, Esengül Öztürk, Fatma Sener, Sema S. Hakki, Özkan Fidan

**Affiliations:** † Department of Bioengineering, Faculty of Life and Natural Sciences, 346448 Abdullah Gul University, Kayseri 38080, Türkiye; ‡ Department of Periodontology, Faculty of Dentistry, 52993 Selcuk University, Konya 42250, Türkiye

**Keywords:** Mesenchymal stem cells, Secreted factors, *Candida albicans*, Cathelicidin (LL-37), Antifungal activity, 3D hydrogel

## Abstract

*Candida albicans* is among the life-threatening fungal species and the primary contributor to hospital-acquired systemic infections, accounting for nearly 70% of all fungal infections worldwide. The current treatment primarily relies on azoles, pyrimidine analogs, polyenes, and echinocandins. However, growing antifungal resistance highlights the urgent need for the development of alternative treatments against *C. albicans*. Mesenchymal stem cells (MSCs) offer huge therapeutic potential for the treatment of *C. albicans*-associated diseases. In this study, palatal adipose tissue-derived MSCs (PAT-MSCs) and PAT-MSCs cultured in 3D biomaterial using nanofibrillar cellulose were tested against *C. albicans* strains ATCC 10231 and ATCC MYA 2876 using an *in vitro* antifungal activity assay. In addition, the conditioned medium from both PAT-MSCs and PAT-MSCs cultured in 3D hydrogel biomaterial (CM-PAT-MSCs-3D) were evaluated for their antifungal activities. The combined effect of PAT-MSCs and their secreted factors was also investigated. The expression of five antimicrobial peptide (AMP)-encoding genes was analyzed by quantitative real-time PCR. The expression of antimicrobial peptides was further confirmed via immunocytochemical staining. PAT-MSCs significantly inhibited the growth of *C. albicans* strains at varying inoculum concentrations (500 and 2000 CFU). Similarly, a comparable antifungal effect was observed when *Candida* strains were treated with PAT-MSC secreted factors alone. Statistical analysis revealed significant differences between the antifungal activities of PAT-MSCs and CM-PAT-MSCs. Lastly, the combination of PAT-MSCs and CM-PAT-MSC-3D led to a marked reduction in fungal growth, with inhibition rates of 99.75% and 99.91% for *C. albicans* ATCC 10231 and ATCC MYA-2876, respectively, at 500 CFU inocula. At 2000 CFU inocula, inhibition rates were 99.54% and 99.91%, respectively (****P ≤ 0.0001). These antifungal activities were further confirmed by using RT-PCR and immunocytochemical analysis. Our findings underscore a perspective on the potent antifungal activity of secreted factors from PAT-MSCs cultured within a 3D hydrogel matrix, specifically against various strains of *C. albicans*. Particularly, the combination of PAT-MSCs with their secreted factors represents a promising therapeutic platform, potentially offering a safer and more effective alternative to conventional antifungal treatments.

## Graphical abstract

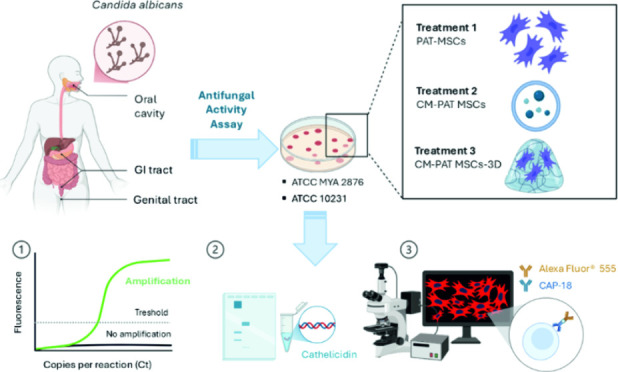

Among the fungal species, only around 600 are considered human pathogens, and *Candida albicans*, an opportunistic pathogen, is classified among the life-threatening fungal species. *Candida* species are reported to be one of the primary organisms associated with systemic infections acquired during hospitalization, accounting for nearly 70% of all fungal infections worldwide. The mortality rate, especially in hospital conditions, is approximately 40%.^1,2^ There are two types of infections caused by *C. albicans*: mucosal infections (affecting the gut, mouth, and vagina) and life-threatening systemic infections (such as candidemia). These infections are primarily caused by a cytolytic peptide toxin known as candidalysin secreted by the hyphal form of *C. albicans*. This toxin plays a critical role in the activation of damage-protection pathways in host cells.^3–5^ *C. albicans* can colonize multiple regions of the body, such as skin, nails, internal organs, and mucous membranes, making it a significant cause of nosocomial infections.^4,6^ Nosocomial infections are notoriously difficult to treat due to the ability of microorganisms to form biofilms, which significantly increase antifungal resistance. *C. albicans* can form biofilms on mucosal surfaces, such as oral mucosa, on medical devices (e.g., voice prostheses and catheters), and in nail infections.^6,7^

Currently, four main classes of drugs are available for *C. albicans* treatment: azoles (e.g., fluconazole), pyrimidine analogs (e.g., 5-flucytosine), polyenes (e.g., nystatin), and echinocandins (e.g., caspofungin). However, growing antifungal resistance has become a major concern. Approximately 7% of patients with *C. albicans*-related infections exhibit resistance to fluconazole, and drug-resistant *Candida* species are responsible for an estimated 1,700 deaths annually.^2,8,9^ Therefore, the development of new therapeutic agents is urgently required to facilitate effective treatment of *C. albicans* associated diseases to overcome the antifungal resistance problem. One promising alternative approach involves the use of mesenchymal stem cells (MSCs) as potential treatments for *C. albicans*-associated diseases.

MSCs have gained significant attention for their therapeutic potential, particularly in reinforcing host immune defenses against bacterial infections.^10–12^ An expanding corpus of research highlights that MSCs can modulate innate immune processes by directly affecting the function of immune effector cells, such as macrophages and neutrophils. This immunoregulatory effect is mediated by the secretion of a variety of bioactive molecules, including prostaglandin (PGE2), interleukins (IL-6, IL-8), and interferon-beta (IFN-β),^13,14^ which collectively contribute to enhanced phagocytic activity and pathogen clearance.^15^ Importantly, MSCs are recognized for their ability to produce antimicrobial peptides (AMPs), including cathelicidin peptide LL37,^16^ hepcidin,^17^ β-defensin 2, and lipocalin 2.^18^ These compounds are typically expressed by neutrophils or epithelial cells, which further enhance immune defense by disrupting microbial membranes and inducing the release of proinflammatory cytokines.^19^ The AMPs secreted by MSCs are also capable of suppressing both bacterial and fungal infections.^12,16,20,21^

Despite their therapeutic attributes, the clinical application of MSCs is hampered by various limitations, such as low engraftment efficiency, potential oncogenic risks, immunogenicity, and concerns related to cell viability and, primarily, safety.^22,23^ To overcome these challenges, researchers have explored cell-free therapeutic alternatives, such as MSC-derived secretomes, capitalizing on the diverse spectrum of biologically active molecules released by stromal cells rather than employing the cells directly. These extracellular vesicles mimic many of the therapeutic effects of parental cells while avoiding the primary risks associated with cell-based therapy.^22,24^ Evidence from preclinical disorder models of human pathological conditions, including cardiovascular, renal, hepatic, pulmonary, and neurodegenerative damage, support the efficacy of MSC-derived exosomes.^25–27^ More recently, the antimicrobial properties of MSCs and their secreted proteins have been confirmed in both experimental and limited clinical settings, with notable outcomes in mouse models of bacterial infections such as methicillin-resistant *Staphylococcus aureus*,^28^ *Escherichia coli*-induced pneumonia,^16^ and *Klebsiella pneumoniae*-induced pneumosepsis.^29^ Furthermore, LL-37 was found to enhance the efficacy of antibiotic-based therapeutic applications in a mouse model of cystic fibrosis.^30^

While substantial evidence supports the antibacterial effects of MSCs, their role in antifungal defense remains insufficiently investigated. A few preliminary studies suggest that MSCs, particularly through the action of LL-37 and its derivatives, can exert antifungal effects against *C. albicans*, possibly through mechanisms that extend beyond membrane permeability disruption and involve intracellular targeting.^21^ An investigation conducted by Cruz and collaborators in 2015 showed that human BM-MSC-derived exosomes can ameliorate airway inflammation resulting from hypersensitivity reactions triggered by *Aspergillus* species in a murine model of steroid-resistant neutrophilic inflammation in severe asthma cases.^31^ Nevertheless, the therapeutic relevance of MSC-derived exosomes in treating fungal infections remains largely unexplored and warrants further scientific inquiry.

In parallel, advancements in tissue engineering have introduced three-dimensional (3D) culture as a tool to reinforce MSCs for therapeutic purposes. It has been shown that 3D culture systems not only facilitate MSC proliferation and differentiation but also enhance their anti-inflammatory functions.^32^ Notably, nanofibrillar cellulose has been identified as a biocompatible scaffold for human MSCs without negatively affecting cell viability.^33^ Furthermore, MSCs cultured within 3D culture secrete elevated levels of multiple growth factors, including hepatocyte growth factor (HGF), vascular endothelial growth factor (VEGF), stromal cell-derived factor-1 (SDF-1), and fibroblast growth factor-2 (FGF-2).^34–36^ Although Toll-like receptors (TLRs) are stimulated by 3D nanofibrillar cellulose and are associated with an enhanced anti-inflammatory response, no prior investigations have explored the antifungal potential of MSCs within 3D cultures, particularly their effects on host immune responses during *Candida* infections in a 3D platform.

Based on these insights, it is hypothesized that MSCs cultured in 3D culture may transfer immunoregulatory molecules, such as cytokines and chemokines, into their exosomes, thereby enhancing host immunity against fungal pathogens. In this context, this study employs nanofibrillar cellulose as a 3D scaffold to stimulate palatal adipose tissue derived-MSCs (PAT-MSCs) and their secreted factors in the cultured medium (CM-PAT-MSCs), with a focus on assessing their antifungal capacity. Specifically, the study evaluates the expression of LL-37, an AMP considered to be a candidate for MSC-mediated antifungal defense, within the 3D microenvironment. These findings offer valuable perspectives on the mechanisms underlying CM-PAT-MSC activity against *C. albicans* and support the development of advanced therapeutic strategies using 3D-cultured PAT-MSCs.

## Results and Discussion

### *In Vitro* Antifungal Activity of PAT-MSCs and Their Secreted Factors

The antifungal potential of PAT-MSCs and their secreted factors was initially evaluated through an *in vitro* fungal killing assay targeting *C. albicans* strains ATCC MYA 2876 and ATCC 10231. The fungal cells were treated with three different experimental setups. They are cocultured either with viable PAT-MSCs or with conditioned media enriched in secreted factors (CM-PAT-MSCs), and their combinations were tested, alongside their corresponding control groups (Figure 1A). This exposure resulted in a pronounced antifungal response, with a greater than 2-log reduction in *Candida* CFU (Figure 1B,C). As expected, the negative control group, containing only culture medium, exhibited no evidence of fungal growth. Conversely, the positive control cohorts in all experimental setups, containing only *C. albicans*, exhibited robust growth compared to the treatment groups (****P ≤ 0.0001). Upon treatment with PAT-MSCs, fungal growth for *C. albicans* ATCC 10231 and *C. albicans* ATCC MYA 2876 was significantly reduced, demonstrating inhibition rates of 98.49% and 99.15% for the 500 CFU inocula, respectively, while the reduction rates were 95.77% and 95.08% for the 2000 CFU inocula (Table S1 and Table S5). Similarly, a comparable antifungal effect was observed when *Candida* strains were treated with CM-PAT-MSCs alone, which also led to a 99.89% and 98.12% reduction in fungal growth of the 500 CFU inocula, and 99.80% and 99.06% inhibition rates were observed in the 2000 CFU inocula (Table S3 and Table S6). Statistical analysis showed a significant difference between the antifungal activity of PAT-MSCs and CM-PAT-MSCs on *Candida* strains (***P ≤ 0.001) (Figure 2). Lastly, the combined effect of PAT-MSCs and their secreted factors was tested. As a result, 99.70% and 99.89% reductions were observed for the 500 CFU inocula, while 98.98% and 99.87% inhibition rates were observed for the 2000 CFU inocula, respectively (****P ≤ 0.0001) (Table 1 and Table S4). Statistical analysis revealed no significant difference in antifungal efficacy between the direct application of CM-PAT-MSCs and their combination (Figure 2).

**Figure 1:**
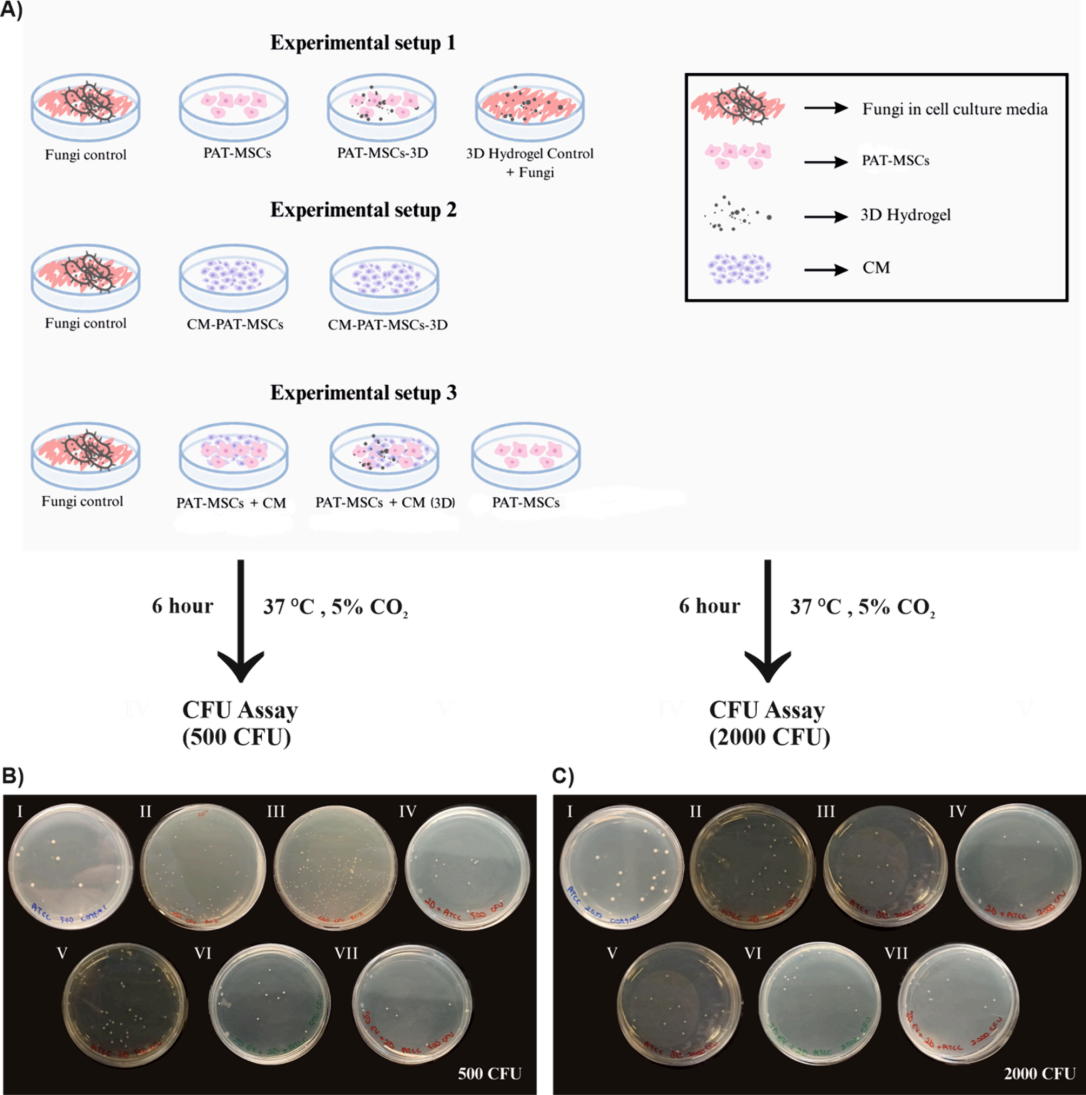
(A) Schematic overview of experimental setups for assessing the antifungal activity of PAT-MSCs, PAT-MSCs-3D, conditioned media (CM), and their combinations against *Candida albicans* strains. (B) Representative PDA plates showing fungal growth of *C. albicans* ATCC 10231 (500 CFU inoculum) under different treatment conditions. (C) Representative PDA plates showing fungal growth of *C. albicans* ATCC 10231 (2000 CFU inoculum) under the same treatment groups. I. Control group - untreated (1:1000 dilution applied prior to plating), II. Treated with PAT-MSCs (1:10 dilution), III. Treated with PAT-MSCs-3D (1:10 dilution for 500 CFU and 1:1000 dilution for 2000 CFU inocula), IV. Treated with CM-PAT-MSCs (1:10 dilution), V. Treated with CM-PAT-MSCs-3D (no dilution), VI. Treated with the combination of PAT-MSCs and CM-PAT-MSCs (no dilution), VII. Treated with the combination of PAT-MSCs and CM-PAT-MSCs-3D (no dilution). The diameters of plates are 60 mm.

**Table 1:** Antifungal Effects of Combined Treatments of PAT-MSCs and CM-PAT-MSCs against *Candida albicans* Strains ATCC 10231 and ATCC MYA 2876 at 500 and 2000 CFU Inocula

*Candida* Strains	Treatment Group	Inoculum Dose (CFU)	% Reduction in CFU vs untreated control^a^
**ATCC MYA 2876**	PAT-MSCs + *Candida*	**500**	97.25
ATCC MYA 2876	PAT-MSCs + CM (3D) + *Candida*	500	99.75
ATCC MYA 2876	PAT-MSCs + CM + *Candida*	500	99.70
ATCC MYA 2876	PAT-MSCs + *Candida*	**2000**	94.14
ATCC MYA 2876	PAT-MSCs + CM (3D) + *Candida*	2000	99.54
ATCC MYA 2876	PAT-MSCs + CM + *Candida*	2000	98.98
**ATCC 10231**	PAT-MSCs + *Candida*	**500**	98.47
ATCC 10231	PAT-MSCs + CM (3D) + *Candida*	500	99.91
ATCC 10231	PAT-MSCs + CM + *Candida*	500	99.89
ATCC 10231	PAT-MSCs + *Candida*	**2000**	99.09
ATCC 10231	PAT-MSCs + CM (3D) + *Candida*	2000	99.91
ATCC 10231	PAT-MSCs + CM + *Candida*	2000	99.87

a Calculated as [(untreated control CFU – treated CFU)/(untreated control CFU)] × 100. Data represent mean values from three biological replicates.

**Figure 2:**
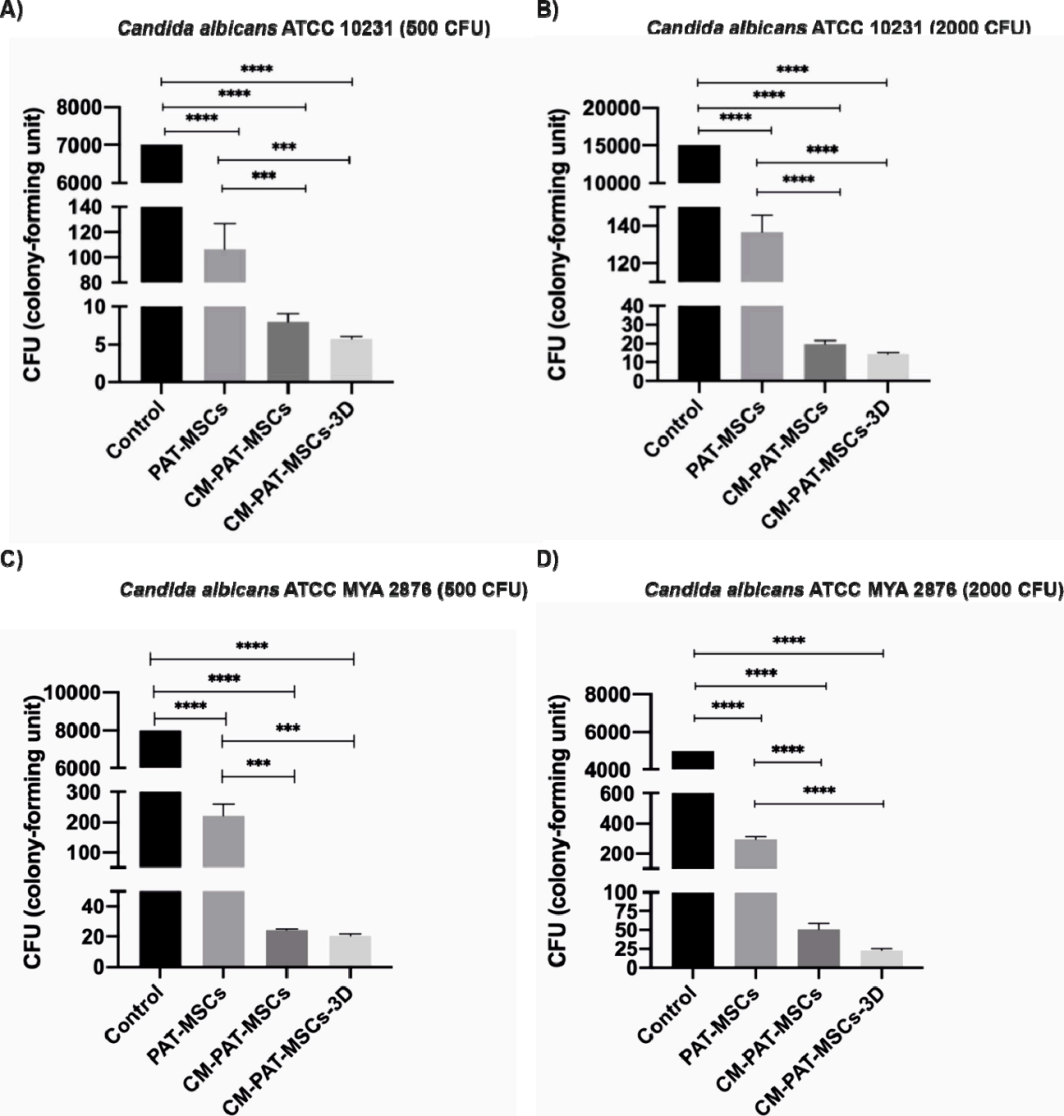
Combination of PAT-MSCs and their secreted factors suppressed the growth of *C. albicans* strains ATCC 10231 and ATCC MYA 2876. (A) Antifungal activity against *C. albicans* ATCC 10231 (500 CFU) following treatment with PAT-MSCs or a combination of PAT-MSCs and CM-PAT-MSCs. (B) Antifungal activity against *C. albicans* ATCC 10231 (2000 CFU) under the same treatment conditions. (C) Antifungal activity against *C. albicans* ATCC MYA 2876 (500 CFU) following treatment with PAT-MSCs or a combination of CM-PAT-MSCs. (D) Antifungal activity against *C. albicans* ATCC MYA 2876 (2000 CFU) under the same treatments. Control: Untreated *C. albicans* strains (ATCC 10231 and ATCC MYA 2876) (*n* = 3 representative images for each group).

### Enhanced Antifungal Efficacy of CM-PAT-MSCs in a 3D Culture Model on *Candida* Infection

To investigate whether PAT-MSCs and their secreted factors exhibit direct antifungal effects against *C. albicans* strains ATCC 10231 and ATCC MYA 2876 within a 3D platform, experiments were performed using a 3D GrowDexT hydrogel model. PAT-MSCs were incubated in the 3D hydrogel for up to 24 h, and their secreted factors were isolated to assess the antifungal efficacy via colony-forming unit (CFU) assay (see experimental setup in Figure 1 and data in Figure 2). First, the antifungal effect of PAT-MSCs cultured in a 3D hydrogel was tested against the *C. albicans* strains ATCC MYA 2876 and ATCC 10231, respectively. Notably, this group did not demonstrate measurable antifungal activity under the experimental conditions (Table S1). In addition, the hydrogel positively affected the fungal growth of *Candida* species compared to control groups, with mean CFU counts of 12,513 and 3,767 for *C. albicans* ATCC 10231 and ATCC MYA 2876 at 500 CFU inocula, while the mean CFU of their control groups was 4,823 and 2,353 under the same conditions, respectively (Table S2). The factors secreted by PAT-MSCs, both independently and when integrated into the 3D hydrogel, exerted a marked antifungal effect, inhibiting fungal growth with the rates of 99.89% and 98.83% for the 500 CFU inocula, while they demonstrated inhibition rates of 99.87% and 99.24% for the 2000 CFU inocula in the 3D platform, respectively (Table S3 and Table S6). Upon treatment with the combination of PAT-MSCs and CM-PAT-MSCs-3D, a significant reduction in fungal growth was observed, with inhibition rates of 99.75% and 99.91% for *C. albicans* ATCC 10231 and ATCC MYA 2876, respectively, for the 500 CFU inocula. For the 2000 CFU inocula, inhibition rates were 99.54% and 99.91%, respectively (****P ≤ 0.0001) (Table 2 and Table S4). Overall, no statistically significant difference was observed between the antifungal efficacy of secreted factors applied under standard conditions and those delivered through the 3D hydrogel (Figure 2). In all experimental setups (except PAT-MSCs cultured in the 3D hydrogel), the untreated *Candida* control groups showed a high fungal burden.

**Table 2:** Primer Sequences Designed for the Selected Genes

Gene	Forward Primer, Reverse Primer (5′ → 3′)
GAPDH:	Fwd 5′-AGC​CAC​ATC​GCT​CAG​ACAC
	Rev 5′-GCC​CAA​TAC​GAC​CAA​ATCC
Beta Defensin 2 (hBD2):	Fwd 5′-CCA​GCC​ATC​AGC​CAT​GAG​GG
	Rev 5′-GGA​GCC​CTT​TCT​GAA​TCC​GC
Cathelicidin (LL37):	Fwd 5′-GAA​GAC​CCA​AAG​GAA​TGG​CC
	Rev 5′-CAG​AGC​CCA​GAA​GCC​TGA​GC
Surfactant Protein D (SPD):	Fwd 5′-ACA​AAA​AGA​AAC​CTG​CCA​TGCT
	Rev 5′-TGG​GCA​TTG​TTC​TGT​GGG​AG
Lipocalin (LCN2):	Fwd 5′-GGA​GCT​GAC​TTC​GGA​ACT​AAA​GG
	Rev 5′-TGT​GGT​TTT​CAG​GGA​GGCC
Hepcidin:	Fwd 5′-CCC​ACA​ACA​GAC​GGG​ACA​AC
	Rev 5′-CTC​CTT​CGC​CTC​TGG​AAC​AT

### Expression of AMPs by CM-PAT-MSCs Cultured in 2D and 3D Platforms

To investigate the transcriptional response of PAT-MSCs to *C. albicans* infection, we examined the mRNA expression of several peptides under both 2D and 3D culture conditions. The AMPs included cathelicidin LL-37, hepcidin, lipocalin (LcN), surfactant protein D (SPD) and beta defensin (hBD2), and were evaluated using reverse transcription quantitative polymerase chain reaction (RT-qPCR), in accordance with protocols adapted from Chow et al. (2020).^38^ The gel electrophoresis images are presented in Figure S1.

Gene expression was quantified and fold change was calculated using the 2^–ΔΔCt^-method,^37^ normalizing target gene expression to the housekeeping gene GAPDH and untreated control samples. As depicted in Figure 3, PAT-MSCs maintained under basal conditions without pathogen exposure did not exhibit detectable expression of the assessed AMPs. In contrast, exposure to *C. albicans* strains triggered upregulation of mRNA expression of AMPs present in the PAT-MSCs in the experimental groups including 2D and 3D culture.

**Figure 3:**
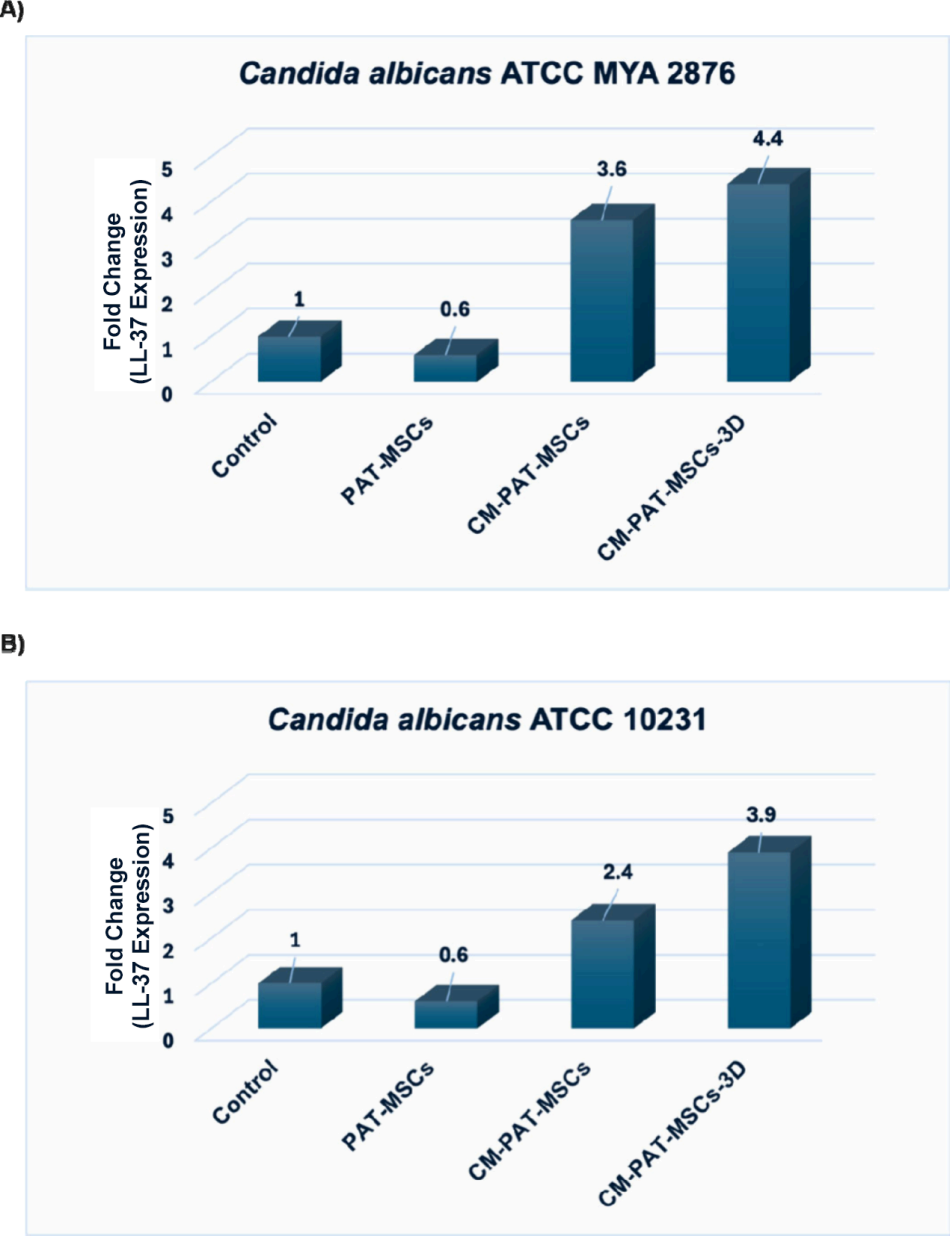
Impact of 3D hydrogel on LL-37 mRNA expression in PAT-MSCs exposed to *C. albicans* strains, as assessed by RT-qPCR. The *y*-axis shows the fold change in gene expression relative to untreated controls and normalized to GAPDH, calculated using the ddCT method. (A) Quantitative representation of LL-37 expression levels against *C. albicans* ATCC MYA 2876. (B) Quantitative representation of LL-37 expression levels against *C. albicans* ATCC 10231. Data points represent the mean of three technical and biological replicates of the PAT-MSCs. *n* ≥ 3 representative images for each group. Control: Only PAT-MSCs without *Candida* strains.

Notably, among the five AMPs analyzed, only LL-37 demonstrated significant mRNA expression in response to fungal stimulation in both culture models (Figure S1). The expression was most pronounced in PAT-MSCs embedded in the 3D GrowDexT hydrogel, indicating enhanced LL-37 gene activation in a 3D environment (Figure 3). The remaining peptides, hepcidin, lipocalin (LcN), beta defensin (hBD2), and surfactant protein D (SPD), showed no detectable mRNA expression across all experimental groups. Consequently, LL-37 was selected for further analysis to explore AMP expression at the functional level.

### Induction of Intracellular LL-37 Expression by CM-PAT-MSCs in a 3D Culture Environment

Previous research has demonstrated that human MSCs are capable of expressing AMPs, including LL-37.^10,16,38^ To validate the transcriptional data obtained through RT-PCR regarding AMP expression, we further assessed the intracellular presence of LL-37 in PAT-MSCs under both 2D and 3D culture conditions following exposure to *C. albicans* ATCC MYA 2876 and ATCC 10231.

Using immunocytochemistry staining with an anti-LL37 antibody, we confirmed that PAT-MSCs actively expressed LL-37 at the intracellular level. Confocal microscopy revealed a significant increase in the LL-37 signal intensity against *C. albicans* ATCC MYA 2876 (Figure 4) and *C. albicans* ATCC 10231 (Figure 5), which was normalized to nuclear DAPI staining. The mean fluorescence intensity of LL-37 was quantified for *C. albicans* ATCC MYA 2876 and *C. albicans* ATCC 10231 (Figure 6). To allow complete assessment of baseline marker expression, representative micrographs for the untreated groups are shown in Figure S2. These findings substantiate the results from gene expression analysis and highlight that 3D culture conditions not only maintain but potentially enhance the functional expression of AMPs such as LL-37 in response to fungal pathogens.

**Figure 4:**
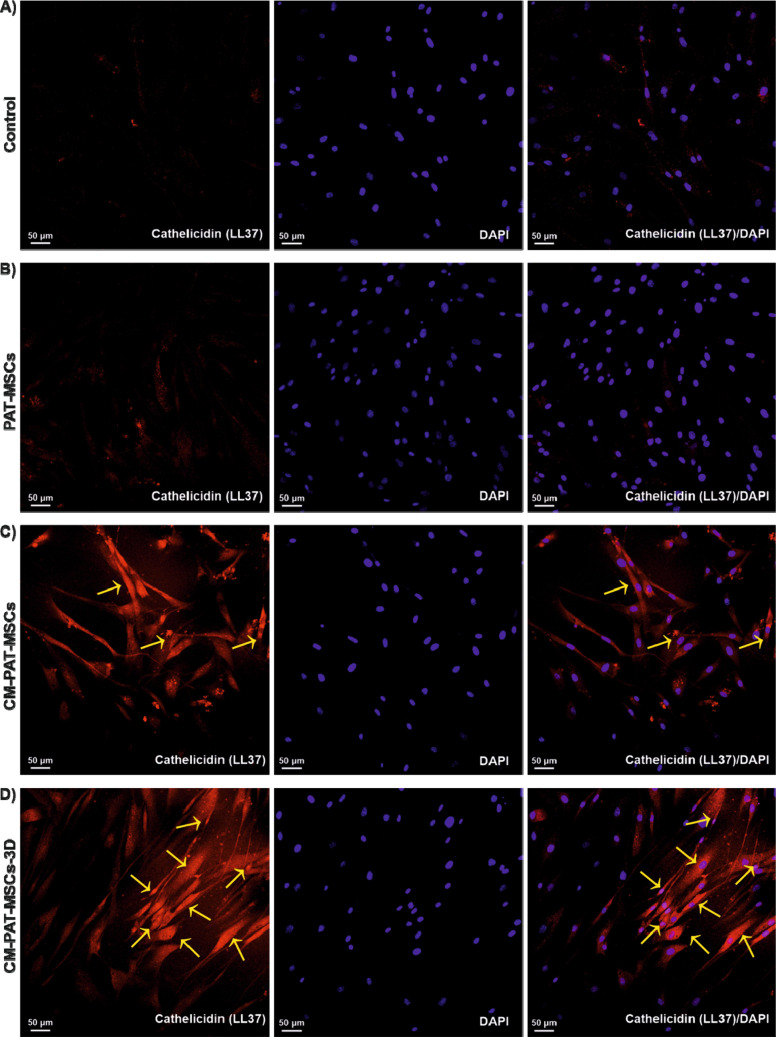
Detection of LL37 expression in CM-PAT-MSCs cultured in 2D and 3D environments by immunocytochemistry. Representative images of PAT-MSCs exposed to *C. albicans* ATCC MYA 2876 and immunostained with anti-LL37 antimicrobial peptide antibody. Specific binding of the antibody to intracellular LL-37 is visualized in a confocal microscope. LL-37 immunostaining shown in red; nuclei counterstained with DAPI (blue). Images were captured at a 20× magnification, scale bar = 50 μm (*n* ≥ 3 representative images for each group). Arrows indicate representative LL-37 expression in red channel. (A) Control: only PAT-MSCs, (B) PAT-MSCs + *C. albicans* ATCC MYA 2876, (C) PAT-MSCs + CM + *C. albicans* ATCC MYA 2876, (D) PAT-MSCs + CM (3D) + *C. albicans* ATCC MYA 2876.

**Figure 5:**
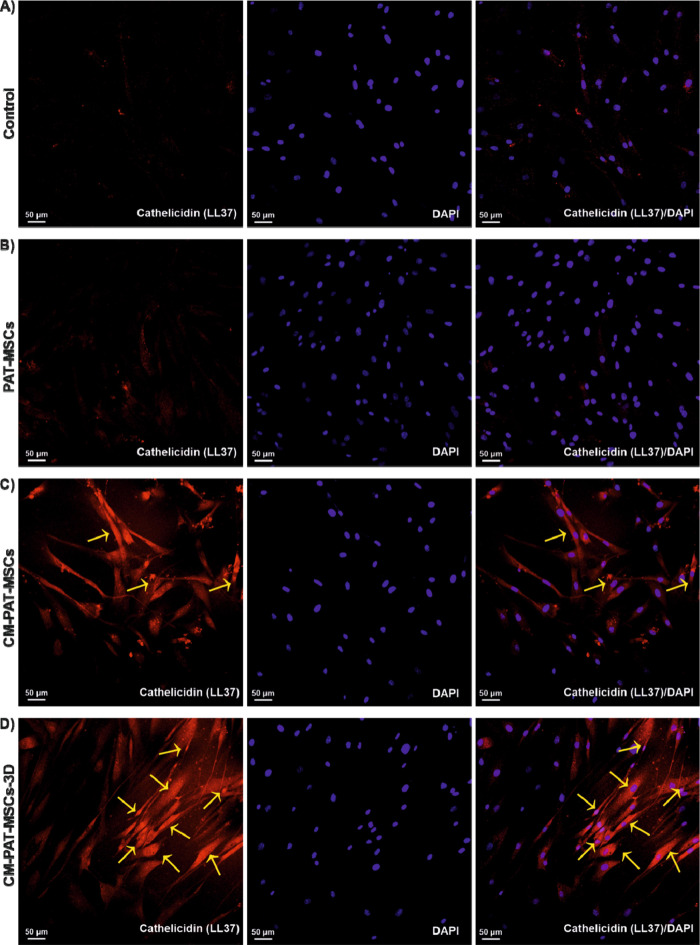
Detection of LL37 expression in CM-PAT-MSCs cultured in 2D and 3D environments by immunocytochemistry. Representative images of PAT-MSCs exposed to *C. albicans* ATCC 10231, immunostained with anti-LL37 antimicrobial peptide antibody. Specific binding of the antibody to intracellular LL-37 is visualized in confocal microscope. LL-37 immunostaining shown in red; nuclei counterstained with DAPI (blue). Images were captured at a 20× magnification, scale bar = 50 μm, (*n* ≥ 3 representative images for each group). Arrows indicate representative LL-37 expression in the red channel. (A) Control: only PAT-MSCs, (B) PAT-MSCs + *C. albicans* ATCC 10231, (C) PAT-MSCs + CM + *C. albicans* ATCC 10231, and (D) PAT-MSCs + CM (3D) + *C. albicans* ATCC 10231.

**Figure 6:**
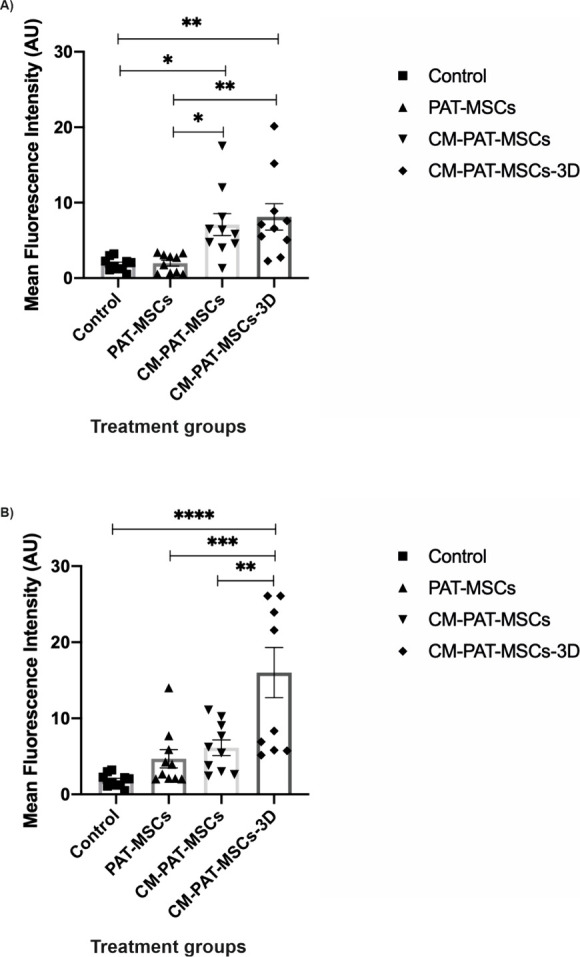
Quantification of mean fluorescence intensity for LL-37 in CM-PAT-MSCs cultured in 2D and 3D systems. (A) Quantitative representation of mean fluorescence intensity for LL-37 expression against *C. albicans* ATCC MYA 2876. (B) Quantitative representation of mean fluorescence intensity for LL-37 expression levels against *C. albicans* ATCC 10231. The *y*-axis represents the mean fluorescence intensity, and the *x*-axis shows treatment groups. Error bars represent SEM. Data are representative of three independent experiments conducted with PAT-MSCs. *n* ≥ 3 representative images for each group. Statistical significance thresholds were defined as follows: ****p ≤ 0.0001, ***p ≤ 0.001, **p ≤ 0.01, *p ≤ 0.05 compared to control (only PAT-MSCs without *Candida* strains).

*C. albicans* is an opportunistic pathogen and can form biofilms on different parts of the body, causing severe diseases. They colonize the mucosal surfaces such as the oral cavity (including dental plaque) and vaginal mucosa.^39^ Particularly, *C. albicans* biofilms were detected in dental plaques from children with severe childhood caries, leading to rampant tooth decay.^39^ This pathogen was also found in endodontic root canals and was associated with persistent or refractory root canal infections.^40^ The infections caused by *Candida* species can lead to death in approximately 40% of cases in immunocompromised individuals and patients receiving immunosuppressive therapy.^41^ The combined treatment with PAT-MSCs and their secreted factors in 3D culture demonstrated a remarkable antifungal effect against *C. albicans* strains ATCC 10231 and ATCC MYA 2876 among all treatment groups. The noticeable reduction in fungal growth at varying inoculum concentrations (500 and 2000 CFU) in different experimental setups shows that the therapeutic potential of the PAT-MSCs and their secreted factors is both effective and consistent under varying conditions. The results also demonstrate the synergistic interactions between PAT-MSCs and CM-PAT-MSCs. In addition, the observed inhibition at higher fungal loads highlights the resilience of the approach, making it a potential alternative or adjunct to conventional antifungal therapies. However, it has been observed that *Candida* species showed higher fungal growth in the presence of 3D hydrogel, contrary to our hypothesis. One possible explanation for this unexpected increase is that the 3D platform may facilitate biofilm formation and induce hyphal development for the *Candida* strains, thereby promoting fungal growth. Alternatively, although cellulose was reported to enhance anti-*Candida* activity in literature, this unexpected result might be due to the utilization of cellulose as a carbon source, potentially facilitated by the expression of carbohydrate-active enzymes such as endo-1,3-β-glucanase encoded in their genome.^42,43^ The CAZy database for carbohydrate-active enzymes does include two strains of *C. albicans*, both of which contain glycoside hydrolases from various families, suggesting a potential for nanofibrillated cellulose degradation by *C. albicans*.^44^ These two potential explanations may account for the enhanced fungal growth observed in the 3D hydrogel condition and the relatively reduced antifungal activity of PAT-MSCs cultured in the 3D hydrogel. In addition, this may also be attributed to the hydrogel providing a protective niche for *Candida albicans*, limiting direct cell–fungus contact or restricting the diffusion of secreted antifungal factors. Moreover, the short coculture duration (6 h) may not have been sufficient for MSCs in 3D to mount an effective response. However, the antifungal activity observed in the conditioned media from PAT-MSC-3D suggests that secreted factors do accumulate and retain activity outside the matrix, supporting the idea that the hydrogel environment influences both the timing and delivery of MSC-mediated antifungal effects.

MSCs have been demonstrated to support the immune response by enhancing the phagocytic activity of the host immune cells and mitigating harmful excessive inflammation, which collectively promotes bacterial clearance.^45,46^ Stem cells isolated easily from palatal tissue exhibit distinct characteristics, including enhanced proliferative capacity and high potency of the regenerative phenotype. This regenerative efficiency is likely attributed either to the presence of specific stem cell populations within the tissue itself or to regenerative biomolecules found in saliva.^47,48^ Due to their potential advantages, palatal adipose tissue can be used as a MSC source. Key antimicrobial peptides (AMPs) such as LL-37 and β-defensin-2 disrupt bacterial membranes and neutralize toxins, while hepcidin restricts iron availability, creating an inhospitable environment for bacterial survival.^10,16^ These peptides, in addition to playing an immunomodulatory role, maintain immune homeostasis and prevent tissue damage from uncontrolled inflammation.^49^ Although limited, studies in murine models suggest that MSCs can inhibit fungal pathogens like *Candida albicans*, potentially through IL-17-mediated mechanisms.^50^ Additionally, MSC-derived secretomes, which include EVs, contribute to antimicrobial defense through both direct pathogen-targeting effects and indirect modulation of the host immune response.^51^

Although preclinical research has highlighted the therapeutic promise of MSC-based interventions in fungal infections, clinical evidence regarding the efficacy of MSC-derived secreted factors remains sparse.^52,53^ Existing studies indicate that fungal pathogenesis is highly complex, and only a limited number of investigations have addressed the antifungal effects of MSCs or their exosomal secretions. One notable study demonstrated that exosomes derived from human bone marrow MSCs alleviated airway inflammation in a mouse model of severe neutrophilic asthma triggered by *Aspergillus* hyphal extract, likely through suppression of Th17 responses.^31^ Moreover, a systematic review found that the MSC-secretome possesses greater antibacterial efficacy than the MSCs themselves.^54^ In parallel, recent findings suggest that stem cells from the human cervix exhibit antifungal activity against diverse *Candida* pathogens including *C. albicans*, *C. krusei*, *C. parapsilosis*, and *C. glabrata*.^55^ Consistent with these observations, current data indicate that the factors secreted by PAT-MSCs exhibited the most significant antifungal activity and their effect was increased by using a 3D culture, particularly against *Candida albicans* strains. The antibacterial action of MSC-derived secretomes has been largely attributed to peptides such as LL-37, beta defensin-2, and hepcidin.^38^ Building on this, this study explored whether the secreted factors derived from PAT-MSCs mediate antifungal effects via similar peptides. Our finding revealed that LL-37 expression was notably upregulated in both 2D and 3D cultures of PAT-MSCs, with 3D culture conditions further enhancing LL-37 gene transcription (Figure 3A,B, Figure 5A,B). In contrast, the expression of other antimicrobial peptides, hepcidin, lipocalin, surfactant protein D, and β-defensin-2, was not detected (Figure S1A). These observations are in line with prior research demonstrating intracellular LL-37 expression in MSCs during bacterial infections. Additionally, significant differences were observed between control and treatment groups, suggesting that the secreted factors derived from PAT-MSCs embedded into 3D hydrogel exhibit superior antifungal activity. This enhancement is likely due to the 3D hydrogel environment promoting LL-37 production against *Candida albicans* strains (Figure 5D). The enhanced transcription of LL-37 in 3D cultures aligns with findings from Chow et al.,^38^ which confirmed the intracellular presence of LL-37 in MSCs challenged with *S. aureus* infections. Collectively, these results suggest that 3D-cultured CM-PAT-MSCs not only have antimicrobial properties themselves but also potentiate the expression and delivery of LL-37, supporting their role as a promising, cell-free therapeutic approach for combating *Candida albicans* infections.

## Conclusions

This study presents novel evidence highlighting the pronounced antifungal efficacy of the factors secreted by PAT-MSCs within a 3D hydrogel matrix, specifically targeting different species of *C. albicans*. Owing to unfavorable effects and constraints linked to traditional therapies including antifungal drugs, the PAT-MSCs in the 3D hydrogel platform emerge as a promising alternative with potential for safer and more effective treatment. However, further investigations are necessary to identify the specific bioactive components, particularly antimicrobial peptides, responsible for this antifungal activity. Future work could confirm the role of LL-37 by measuring LL-37 protein levels in the CM or testing whether an LL-37 neutralizing antibody or inhibitors reduce the antifungal activity. If substantiated by future research, these findings may pave the way for innovative therapeutic strategies against drug-resistant *Candida* infections.

## Experimental Section

### Isolation and Characterization of PAT-MSCs

Ethical approval for the isolation of PAT-MSCs was received from the Ethics Committee of the Faculty of Dentistry, Selcuk University, and the Committee of the University of Kocaeli.^56^ Tissue samples were collected from patients in accordance with the Declaration of Helsinki, and written informed consent was obtained from all participants. MSCs were isolated from a 1 × 1 mm^2^ segment of palatal adipose tissue located within connective tissue grafts collected during surgical procedures for gingival recession. The donor groups were two systemically selected periodontally healthy female individuals, aged 25 and 30 years, selected based on specific inclusion criteria. The isolated MSCs were identified and validated according to the criteria proposed by the International Society for Cellular Therapy.^57^ For characterization of MSCs, flow cytometric analysis, immunofluorescence staining, and trilineage differentiation assays were performed.^56^

### Cell Culture of PAT-MSCs

Human PAT-MSCs were cultured in Dulbecco’s Modified Eagle Medium (DMEM), supplemented with l-glutamine (2 mM), penicillin (100 units/mL), streptomycin 100 μg/mL), and 10% (v/v) heat-inactivated fetal bovine serum (FBS) (all reagents sourced from Sigma-Aldrich, Gillingham, UK). Additionally, basic fibroblast growth factor (5 ng/mL bFGF; Peprotech, UK) was included to promote proliferation. The cultures were incubated at 37 °C in a humidified atmosphere containing 5% CO_2_ using a BINDER APT.line C150 incubator. The culture medium was refreshed every 2–3 days, and cells were passaged until they reached 70% confluence.

For the 3D culture, nanofibrillated cellulose was purchased from UPM Biochemicals (Helsinki, Finland). The preparation of the GrowDexT hydrogel-based biomaterial at a final concentration of 0.2% was conducted as previously described.^33^ Then, PAT-MSCs were enzymatically dissociated using 0.05% trypsin/EDTA (Sigma-Aldrich) and subsequently combined with 1% w/v stock solution of 3D GrowDexT hydrogel to formulate a final concentration of 0.2% (w/v). The cells were embedded at a density of 1 × 10^5^ cells/mL within the 3D GrowDexT hydrogel and cultivated in 24-well cell culture plates.^33^ All experiments were conducted using PAT-MSCs between passages 7 and 11. To ensure consistency across biological replicates, PAT-MSCs within a range of 3 passages were used.

### Preparation of CM-PAT-MSCs and CM-PAT-MSCs-3D

Human PAT-MSCs were expanded in DMEM medium supplemented with 2 mM l-glutamine, 100 U/mL penicillin, and 100 μg/mL streptomycin (all from Sigma-Aldrich, Gillingham, UK), enriched with 10% v/v heat-inactivated FBS and 5 ng/mL bFGF to obtain standard medium. The MSC seeding concentration was 1 × 10^5^ cells/mL in 24-well plates. The cells were incubated at 37 °C with 5% CO_2_ under humidified conditions until reaching 70% confluence. A phosphate-buffered saline (PBS) rinse was performed to cleanse the cells of residual substances, and then cells were transferred to fresh DMEM containing 10% FBS and incubated for an additional 48 h. Subsequently, PBS was employed to wash the cells three times, and cells were cultured again in serum-free DMEM. After 48 h, the medium was harvested and centrifuged at 300*g* for 5 min. The cell-free liquid phase containing the secreted factors was harvested from low-passage cells (passage 1–2), subsequently freeze-dried, reconstituted in deionized distilled water, and stored at −80 °C for later experimental applications.^58^

For the isolation of PAT-MSCs cultured in 3D hydrogel, PAT-MSCs were embedded into 3D GrowDexT hydrogel at a final concentration of 0.2% w/v by mixing cell suspensions with 1% w/v stock, yielding a cell density of 1 × 10^5^ cells/mL. After reaching 70% confluence, cells were rinsed with PBS and recultured in DMEM with 10% FBS in 5% CO_2_ at 37 °C for 48 h. Following this, the cultures were washed three times with PBS and incubated again in serum-free DMEM. After an additional 48 h, the medium was centrifuged at 300*g* for 5 min. The collected supernatant, enriched with the secreted factors, was harvested from passage 1 to 2, freeze-dried, reconstituted in deionized distilled water, and stored at −80 °C until later experimental applications.

### *Candida* Strains and Growth Conditions

*C. albicans* ATCC 10231 and *C. albicans* ATCC MYA 2876, two opportunistic *Candida* strains, were selected for antifungal activity testing. The strains were cultured in Potato Dextrose Broth (PDB) (Conda Lab) following a 16 h incubation at 37 °C, and the culture conditions were then modified to 500 and 2000 colony-forming units (CFU)/mL based on optical density measurements at 600 nm (OD_600nm_).

### Antifungal Activity Assay

The experimental setup comprised four groups: (1) Group 1, fungal controls (growth medium, antibiotic-free, inoculated with fungi); (2) Group 2, PAT-MSCs cocultured with fungi; (3) Group 3, PAT-MSCs cultured in 3D hydrogel inoculated with fungi; (4) Group 4, 3D hydrogel alone in cell culture media inoculated with fungi (control) (Figure 1). For each group, fungal cultures adjusted to the required CFU concentrations were combined with 400 μL of cell culture medium containing PAT-MSCs or PAT-MSCs cultured in a 3D hydrogel. The cell densities of PAT-MSCs were set to 1 × 10^5^. Samples were maintained for 6 h at 37 °C in a humidified incubator with 5% CO_2_. Following incubation, a 50 μL portion from each well was plated onto PDB agar. After overnight incubation at 37 °C, the fungal colonies were manually counted. The results were compared to the fungal controls (Group 1) and expressed as percentage values. All experiments were performed in triplicate.

The same process was performed using factors secreted by PAT-MSCs. In this case, a previously established protocol was followed with minor modifications.^28^ A series of defined experimental conditions comprised three groups: (1) Group 1, fungal controls (growth medium, antibiotic-free, inoculated with fungi); (2) Group 2, secreted factors from PAT-MSCs in the culture medium inoculated with fungi; (3) Group 3, secreted factors from PAT-MSCs cultured in 3D hydrogel culture medium inoculated with fungi (Figure 1). For each group, 100 μL of PDB was combined with 100 μL of the corresponding secreted factors. Samples were incubated for 6 h at 37 °C in a humidified incubator with 5% CO_2_. Following the incubation period, a 50 μL portion from each well was plated onto PDB agar. After overnight incubation at 37 °C, the colonies were manually counted. The results were compared to the fungal controls (Group 1) and expressed as percentage values. All samples were performed in triplicate.

Subsequently, the combined effect of the secreted factors and PAT-MSCs was tested. In this section, four experimental groups were evaluated: (1) Group 1, fungal controls (growth medium, antibiotic-free, inoculated with fungi); (2) Group 2, CM-PAT-MSCs combined with PAT-MSCs, inoculated with fungi; (3) Group 3, CM-PAT-MSCs cultured in 3D hydrogel combined with PAT-MSCs, inoculated with fungi; (4) Group 4, only PAT-MSCs, inoculated with fungi (MSC control) (Figure 1). For each experimental group, 100 μL of the secreted factors was mixed with 400 μL of the corresponding PAT-MSC sample. The mixtures were incubated for 6 h at 37 °C with 5% CO_2_. Following the incubation period, a 50 μL portion from each well was plated onto PDB agar. After overnight incubation at 37 °C, the colonies were manually counted, and the results were compared to the fungal controls (Group 1) and expressed as percentage values. Each experimental condition was performed in triplicate to validate the consistency of the results.

### RNA Extraction and Quantitative Real-Time PCR (RT-qPCR) Analysis

To assess the transcriptional expression of AMPs produced by PAT-MSCs in 2D and 3D, cells were seeded at a density of 1 × 10^5^ cells/mL in 24-well culture plates and incubated at 37 °C and 5% CO_2_ for 24 h. Negative controls were also maintained under identical conditions. For experimental coculture with *C. albicans*, two inoculum concentrations (500 CFU and 2000 CFU) were prepared in 50 μL aliquot and introduced to the wells. A 6 h incubation was then performed at 37 °C in a 5% CO_2_ incubator. Unless specified otherwise, culture medium prepared without antibiotics was used throughout the study.

Total RNA was extracted using TransZol (ET101-01, Transgen Biotech) following the manufacturer’s protocol. RNA concentrations were quantified using a NanoDrop ND-1000 spectrophotometer. Subsequently, 1 μg of RNA was reverse transcribed into complementary DNA (cDNA) utilizing the Easy Script First Strand cDNA Synthesis Kit (AE301, Transgen Biotech). Quantitative PCR was conducted using gene-specific primers designed via Primer-BLAST (NCBI), with final primer concentrations of 100 (forward) and 200 nM (reverse). Amplification was detected with a TransStart Green qPCR Supermix (AQ101), and fluorescence data were collected for expression analysis. Relative gene expression fold change was determined using the delta delta Ct (ddCT) method with normalization against housekeeping gene GAPDH and untreated control. Relative gene expression levels were calculated utilizing the comparative 2^–ΔΔCt^-method.^37^ The specific oligonucleotide primers utilized for gene expression are provided in Table 2.^38^

### Immunocytochemical Staining

To determine intracellular expression of AMPs produced by PAT-MSCs in 2D and 3D culture, the prepared cells on coverslips were fixed with 4% paraformaldehyde (PFA) for 10 min. After being washed with PBS, the cells were permeabilized with 0.1% Triton X-100 to facilitate antibody penetration. To reduce nonspecific binding, samples were incubated in 5% (v/v) normal donkey serum to prevent nonspecific antibody binding and then were incubated with primary antibodies.

Based on prior RT-qPCR results and subsequent statistical analysis, one or more of the specified primary AMP antibodies would be selected to perform immunocytochemical staining, focusing on peptides with significant expression in treated experimental groups. Specifically, the Anti-cathelicidin antibody (CAMP/Cathelicidin antimicrobial peptide antibody, FINETEST, FNab01241) was applied at a dilution of 1:100.^38^ Following a 60 min incubation with the primary antibody, the samples were washed thoroughly and incubated for an additional 60 min with a secondary antibody (Alexa Fluor 555 Donkey anti-rabbit IgG Antibody, BioLegend, 406412/100 μg), diluted at 1:300. Nuclear staining was performed using 4′,6-diamidino-2-phenylindole (DAPI) for 20 min. Fluorescent signals were visualized by using confocal laser scanning microscopy.

### Statistical Analyses

To compare two independent groups, the nonparametric Mann–Whitney test was applied. For analyses consisting of more than two groups, one-way ANOVA was conducted, followed by Tukey’s post hoc test to determine significant differences among groups. To assess potential synergistic interactions, a two-way ANOVA was utilized. A statistically significant interaction term (*P* ≤ 0.05) was interpreted as evidence of synergy.^59^ Synergistic efficacies were defined as a statistically significant increment in the mean fungal killing efficiency observed when antibiotics were combined with CM-PAT-MSCs, relative to antibiotics alone or PAT-MSC alone. All statistical computations were performed by using Prism 8 software (GraphPad, La Jolla, California). Statistical significance thresholds were defined as follows: *p ≤ 0.05, **p ≤ 0.01, ***p ≤ 0.001, and ****p ≤ 0.0001.

## Supplementary Material



## Data Availability

Data supporting this study are included within the article.

## Notes

Antifungal effects of PAT-MSCs and PAT-MSCs-3D treatments against *Candida albicans* strains ATCC MYA 2876 and ATCC 10231 (Table S1); Effect of 3D hydrogel alone on fungal growth of *Candida albicans* strains ATCC MYA 2876 and ATCC 10231 at 500 CFU inocula (Table S2); Antifungal effects of CM-PAT-MSCs and CM-PAT-MSCs-3D treatments against *Candida albicans* strains ATCC MYA 2876 and ATCC 10231 (Table S3); Antifungal effects of combined treatments of PAT-MSCs and CM-PAT-MSCs against *Candida albicans* strains ATCC MYA 2876 and ATCC 10231 (Table S4); Killing percentages of PAT-MSCs and PAT-MSCs-3D treatments against *Candida albicans* strains ATCC MYA 2876 and ATCC 10231 (Table S5); Killing percentages of CM-PAT-MSCs and CM-PAT-MSCs-3D treatments against *Candida albicans* strains ATCC MYA 2876 and ATCC 10231 (Table S6); Gel electrophoresis images (Cropped Gel) (Figure S1); Representative images of untreated groups (Figure S2) (PDF)
