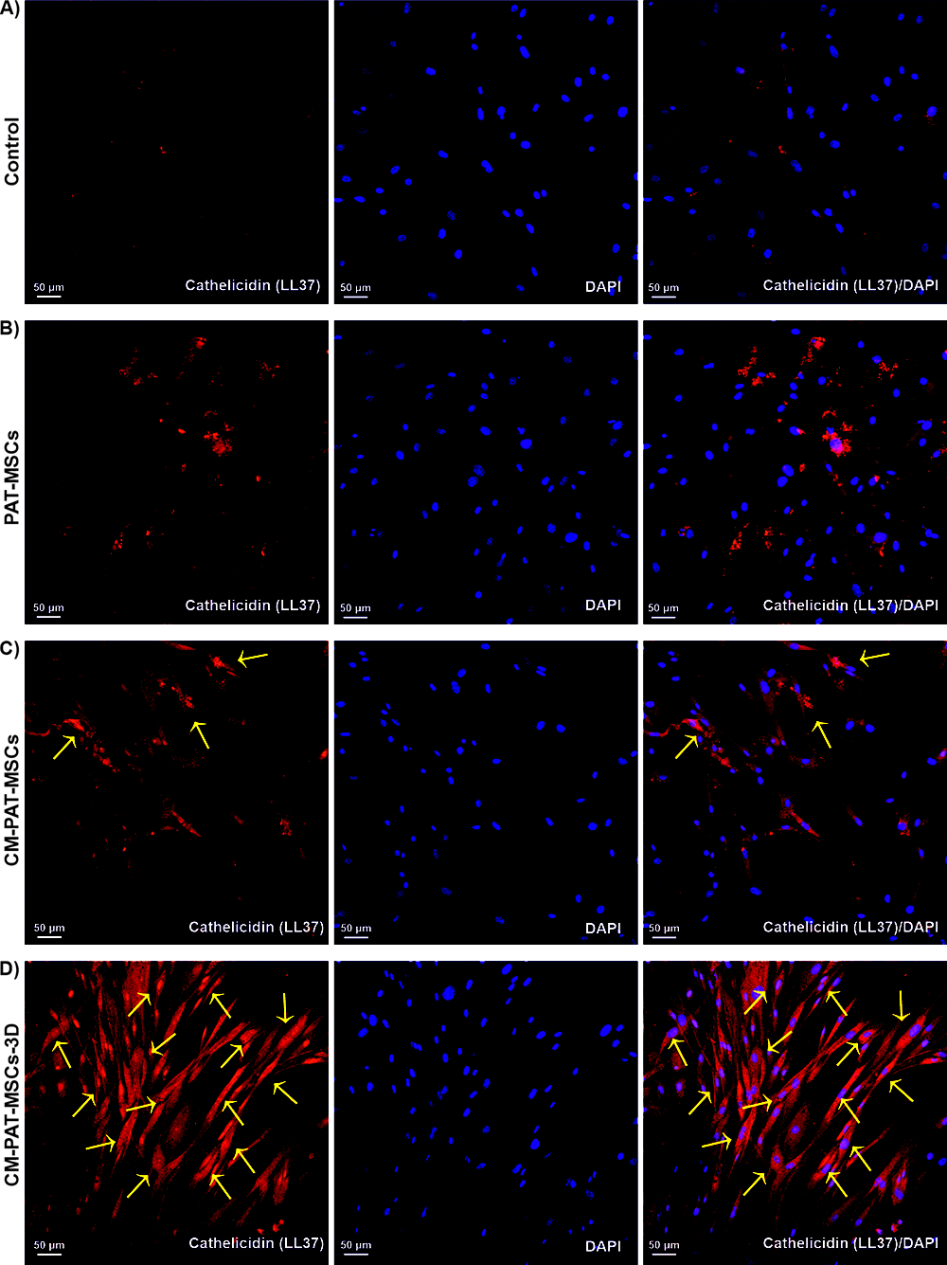# Correction to “Antifungal Efficacy of 3D-Cultured Palatal Mesenchymal Stem Cells and Their Secreted Factors against *Candida albicans*”

**DOI:** 10.1021/acsinfecdis.5c01127

**Published:** 2026-01-03

**Authors:** Mesude Bicer, Esengül Öztürk, Fatma Sener, Sema S. Hakki, Özkan Fidan

In the originally published article, Figure 5 (page number: 2901) was inadvertently replaced with a duplicate of Figure 4 during manuscript revision. The correct version of Figure 5 has now been updated and is below. This correction does not affect the results or conclusions of the study.

**Figure 5:**