# Supplementary material for: Antifungal Efficacy of 3D-Cultured Palatal Mesenchymal Stem Cells and Their Secreted Factors against Candida albicans
Source: ACS Infect Dis. 2025 Sep 19;11(10):2894–906. doi: 10.1021/acsinfecdis.5c00657 (PMC12519483; doi:10.1021/acsinfecdis.5c00657)
Supplement: Supplementary file 1 [file id5c00657_si_001.pdf]

Supporting Information

Antifungal Efficacy of 3D-Cultured Palatal Mesenchymal Stem Cells and Their Secreted Factors Against *Candida albicans*

Mesude Bicer [1,*], Esengül Öztürk [1,#], Fatma Sener [1,#], Sema S. Hakki [2], Özkan Fidan [1]

[1] Department of Bioengineering, Faculty of Life and Natural Sciences, Abdullah Gul University, Kayseri, 38080, Türkiye
[2] Department of Periodontology, Faculty of Dentistry, Selcuk University, Konya, 42250, Türkiye

[#]These authors contribute equally.
[*] Correspondence: mesude.bicer@agu.edu.tr

Table S1: Antifungal effects of PAT-MSCs and PAT-MSCs-3D treatments against *Candida albicans* strains ATCC MYA 2876 and ATCC 10231

Candida Strains	Inoculum Dose (CFU)	PAT-MSCs (CFU)	PAT-MSCs-3D (CFU)	Control Group (CFU)
**ATCC MYA 2876**	500	80<br>68<br>70<br>**Mean:** 72.6<br>**SD:** 6.4	518<br>180<br>486<br>**Mean:** 394.6<br>**SD:** 186.5	3710<br>5800<br>4960<br>**Mean:** 4823.3<br>**SD:** 1051.6
**ATCC MYA 2876**	2000	610<br>960<br>670<br>**Mean:** 746.6<br>**SD:** 187.1	2400<br>3580<br>2080<br>**Mean:** 2686.6<br>**SD:** 790.0	19000<br>19000<br>15000<br>**Mean:** 17666.6<br>**SD:** 2309.4
**ATCC 10231**	500	8<br>18<br>34<br>**Mean:** 20.0<br>**SD:** 13.1	212<br>78<br>76<br>**Mean:** 122.0<br>**SD:** 77.9	2220<br>2570<br>2270<br>**Mean:** 2353.3<br>**SD:** 189.2
**ATCC 10231**	2000	670<br>410<br>150<br>**Mean:** 410<br>**SD:** 260	460<br>1430<br>1310<br>**Mean:** 1066.6<br>**SD:** 528.8	7000<br>10000<br>8000<br>**Mean:** 8333.3<br>**SD:** 1527.5

Table S2: Effect of 3D hydrogel alone on fungal growth of *Candida albicans* strains ATCC MYA 2876 and ATCC 10231 at 500 CFU inocula

Candida Strains	Inoculum Dose (CFU)	3D Hydrogel + *Candida* (CFU)	Cell Culture Medium + *Candida* (CFU)
**ATCC MYA 2876**	500	12500<br>12520<br>12520<br>**Mean:** 12513.3<br>**SD:** 11.5	3710<br>5800<br>4960<br>**Mean:** 4823.3<br>**SD:** 1051.6
**ATCC 10231**	500	3800<br>3800<br>3700<br>**Mean:** 3766.6<br>**SD:** 57.7	2220<br>2570<br>2270<br>**Mean:** 2353.3<br>**SD:** 189.2

Table S3: Antifungal effects of CM-PAT-MSCs and CM-PAT-MSCs-3D treatments against *Candida albicans* strains ATCC MYA 2876 and ATCC 10231

Candida Strains	Inoculum Dose (CFU)	CM-PAT-MSCs (CFU)	CM-PAT-MSCs-3D (CFU)	Control Group (CFU)
**ATCC MYA 2876**	500	820<br>630<br>730<br>**Mean:** 726.6<br>**SD:** 95.0	530<br>850<br>680<br>**Mean:** 686.6<br>**SD:** 160.1	610000<br>690000<br>627000<br>**Mean:** 642333.3<br>**SD:** 42146.5
**ATCC MYA 2876**	2000	1200<br>1600<br>1700<br>**Mean:** 1500.0<br>**SD:** 264.5	1000<br>1200<br>700<br>**Mean:** 966.6<br>**SD:** 251.6	520000<br>1050000<br>720000<br>**Mean:** 763333.3<br>**SD:** 267644.0
**ATCC 10231**	500	280<br>610<br>630<br>**Mean:** 506.6<br>**SD:** 196.5	350<br>250<br>250<br>**Mean:** 283.3<br>**SD:** 57.7	29000<br>28000<br>24000<br>**Mean:** 27000.0<br>**SD:** 2645.7
**ATCC 10231**	2000	1700<br>2800<br>1700<br>**Mean:** 2066.6<br>**SD:** 635.0	2200<br>1700<br>1100<br>**Mean:** 1666.6<br>**SD:** 550.7	160000<br>150000<br>350000<br>**Mean:** 220000.0<br>**SD:** 112694.2

Table S4: Antifungal effects of combined treatments of PAT-MSCs and CM-PAT-MSCs against *Candida albicans* strains ATCC MYA 2876 and ATCC 10231

Candida Strains	Inoculum Dose (CFU)	PAT-MSCs (CFU)	PAT-MSCs + CM (CFU)	PAT-MSCs + CM (3D) (CFU)	Control Group (CFU)
**ATCC MYA 2876**	500	290<br>220<br>150<br>**Mean:** 220.0<br>**SD:** 70.0	25<br>25<br>23<br>**Mean:** 24.3<br>**SD:** 1.1	23<br>20<br>18<br>**Mean:** 20.3<br>**SD:** 2.5	8000<br>8000<br>8000<br>**Mean:** 8000.0<br>**SD:** 0.0
**ATCC MYA 2876**	2000	300<br>320<br>260<br>**Mean:** 293.3<br>**SD:** 30.5	60<br>58<br>34<br>**Mean:** 50.6<br>**SD:** 14.4	23<br>27<br>19<br>**Mean:** 23.0<br>**SD:** 4.0	5000<br>5000<br>5000<br>**Mean:** 5000.0<br>**SD:** 0.0
**ATCC 10231**	500	140<br>70<br>110<br>**Mean:** 106.6<br>**SD:** 35.1	9<br>6<br>9<br>**Mean:** 8.0<br>**SD:** 1.7	6<br>6<br>5<br>**Mean:** 5.6<br>**SD:** 0.5	7000<br>7000<br>7000<br>**Mean:** 7000.0<br>**SD:** 0.0
**ATCC 10231**	2000	120<br>140<br>150<br>**Mean:** 136.6	23<br>18<br>18<br>**Mean:** 19.6	16<br>14<br>13<br>**Mean:** 14.3	15000<br>15000<br>15000

		**SD:** 15.2	**SD:** 2.8	**SD:** 1.4	**Mean:** 15000.0 **SD:** 0.0

Table S5: Killing percentages of PAT-MSCs and PAT-MSCs-3D treatments against *Candida albicans* strains ATCC MYA 2876 and ATCC 10231

***Candida* Strains**	**Inoculum Dose (CFU)**	**Treatment Groups**	**% Reduction in CFU vs untreated control***
**ATCC MYA 2876**	500	PAT-MSCs	98.49%
**ATCC MYA 2876**	500	PAT-MSCs-3D	91.81%
**ATCC MYA 2876**	2000	PAT-MSCs	95.77%
**ATCC MYA 2876**	2000	PAT-MSCs-3D	84.79%
**ATCC 10231**	500	PAT-MSCs	99.15%
**ATCC 10231**	500	PAT-MSCs-3D	94.81%
**ATCC 10231**	2000	PAT-MSCs	95.08%
**ATCC 10231**	2000	PAT-MSCs-3D	87.20%

*** Calculated as [(untreated control CFU - treated CFU) / untreated control CFU] x 100**

Table S6: Killing percentages of CM-PAT-MSCs and CM-PAT-MSCs-3D treatments against *Candida albicans* strains ATCC MYA 2876 and ATCC 10231

***Candida* Strains**	**Inoculum Dose (CFU)**	**Treatment Groups**	**% Reduction in CFU vs untreated control***
**ATCC MYA 2876**	500	CM-PAT-MSCs	99.89%
**ATCC MYA 2876**	500	CM-PAT-MSCs-3D	99.89%
**ATCC MYA 2876**	2000	CM-PAT-MSCs	99.80%
**ATCC MYA 2876**	2000	CM-PAT-MSCs-3D	99.87%
**ATCC 10231**	500	CM-PAT-MSCs	98.12%
**ATCC 10231**	500	CM-PAT-MSCs-3D	98.83%
**ATCC 10231**	2000	CM-PAT-MSCs	99.06%
**ATCC 10231**	2000	CM-PAT-MSCs-3D	99.24%

*** Calculated as [(untreated control CFU - treated CFU) / untreated control CFU] x 100**

A)

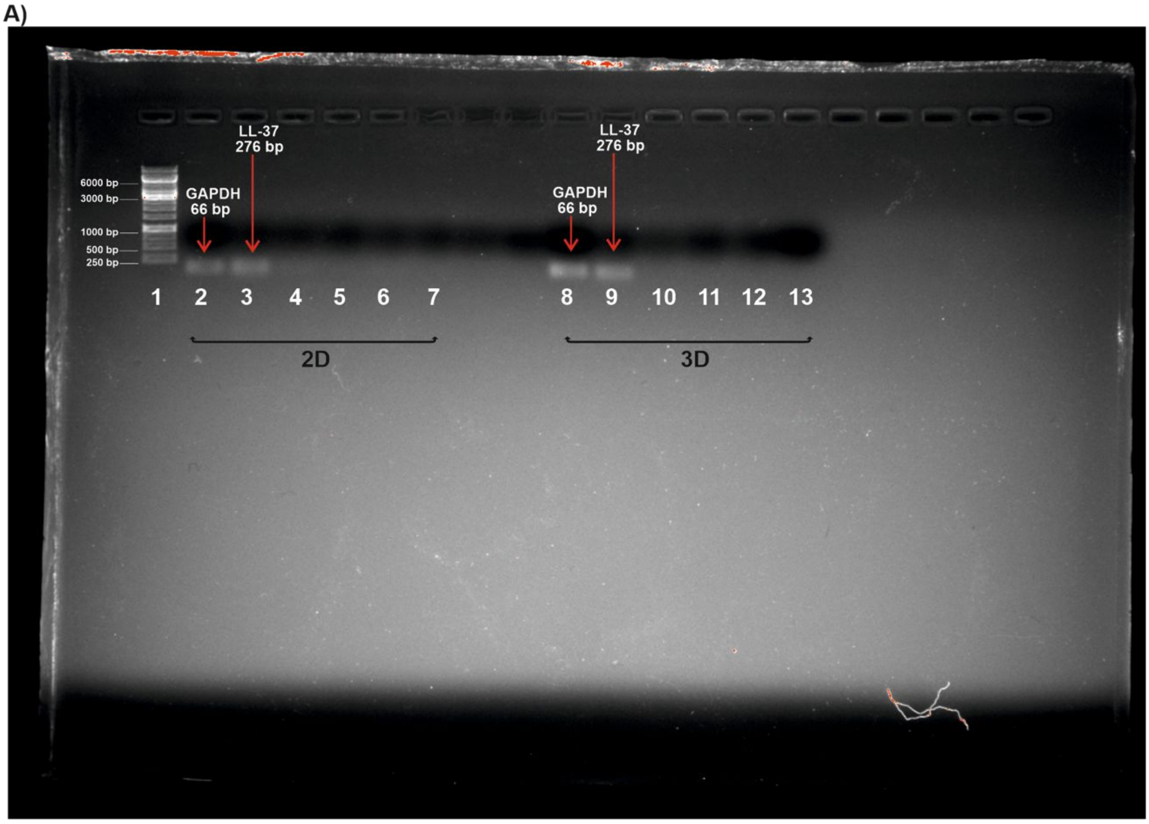

B)

C)

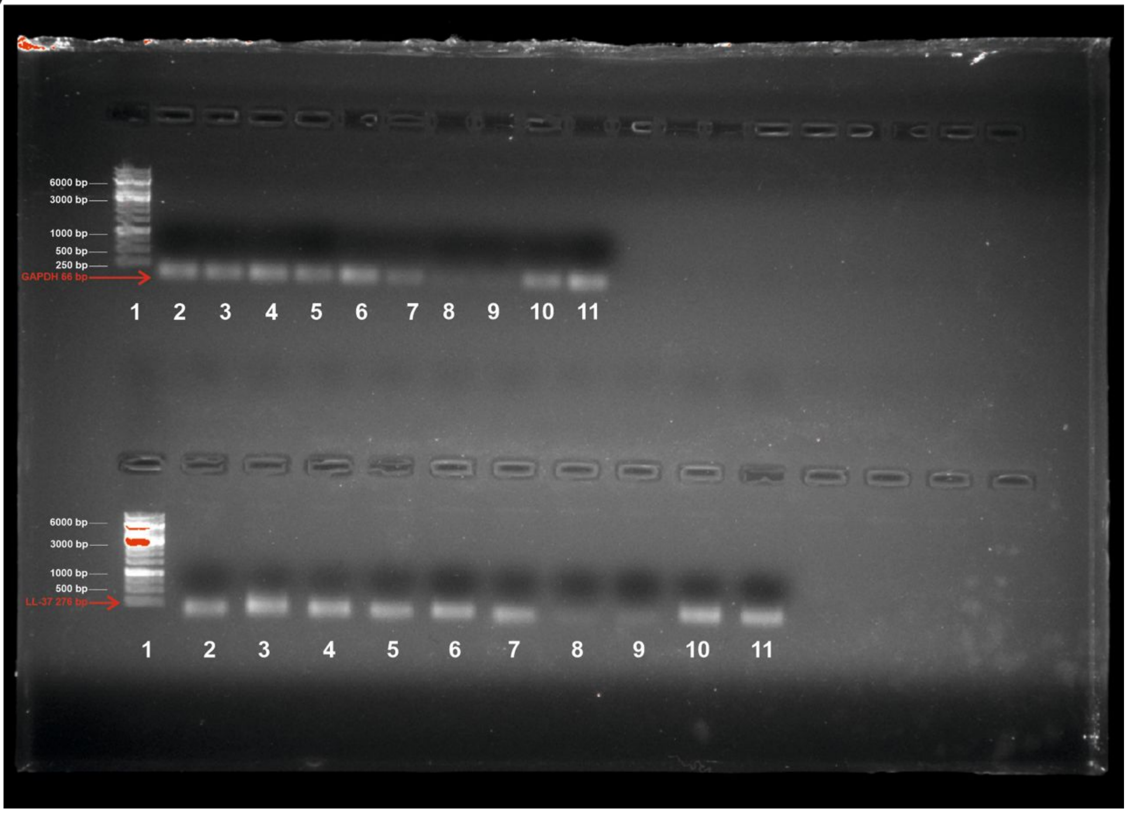

Figure S1. Gel electrophoresis images (Cropped Gel). Lane 1 is designated for the DNA ladder, serving as a molecular weight marker for size calibration. (A) Lanes 2-7 are used to display PAT-MSCs cultured in 2D culture

for the presence of GAPDH (66 bp), LL37 (276 bp), Beta-Defensin (255 bp), Hepcidin (93 bp), Lipocalin (71 bp), Surfactant Protein D (114 bp). Lanes 8-13 represent PAT-MSCs cultured in 3D culture for the presence of GAPDH, LL37, Beta-Defensin, Hepcidin, Lipocalin, Surfactant Protein D. (B) Lanes 2-11 represent PAT-MSCs expanded in 2D and 3D culture for the presence of GAPDH. (C) Lanes 2-11 represent PAT-MSCs cultured in 2D and 3D culture for the presence of LL37. Lane 2:PAT-MSCs untreated, Lane 3:PAT-MSCs + *C. albicans* ATCC MYA 2876, Lane 4:PAT-MSCs + *C. albicans* ATCC 10231, Lane 5:CM-PAT-MSCs + *C. albicans* ATCC MYA 2876, Lane 6:CM-PAT-MSCs + *C. albicans* ATCC 10231, Lane 7:PAT-MSCs in 3D culture untreated, Lane 8:PAT-MSCs in 3D + *C. albicans* ATCC MYA 2876, Lane 9:PAT-MSCs in 3D + *C. albicans* ATCC 10231, Lane 10:CM-PAT-MSCs in 3D + *C. albicans* ATCC MYA 2876, Lane 11:CM-PAT-MSCs in 3D + *C. albicans* ATCC 10231.

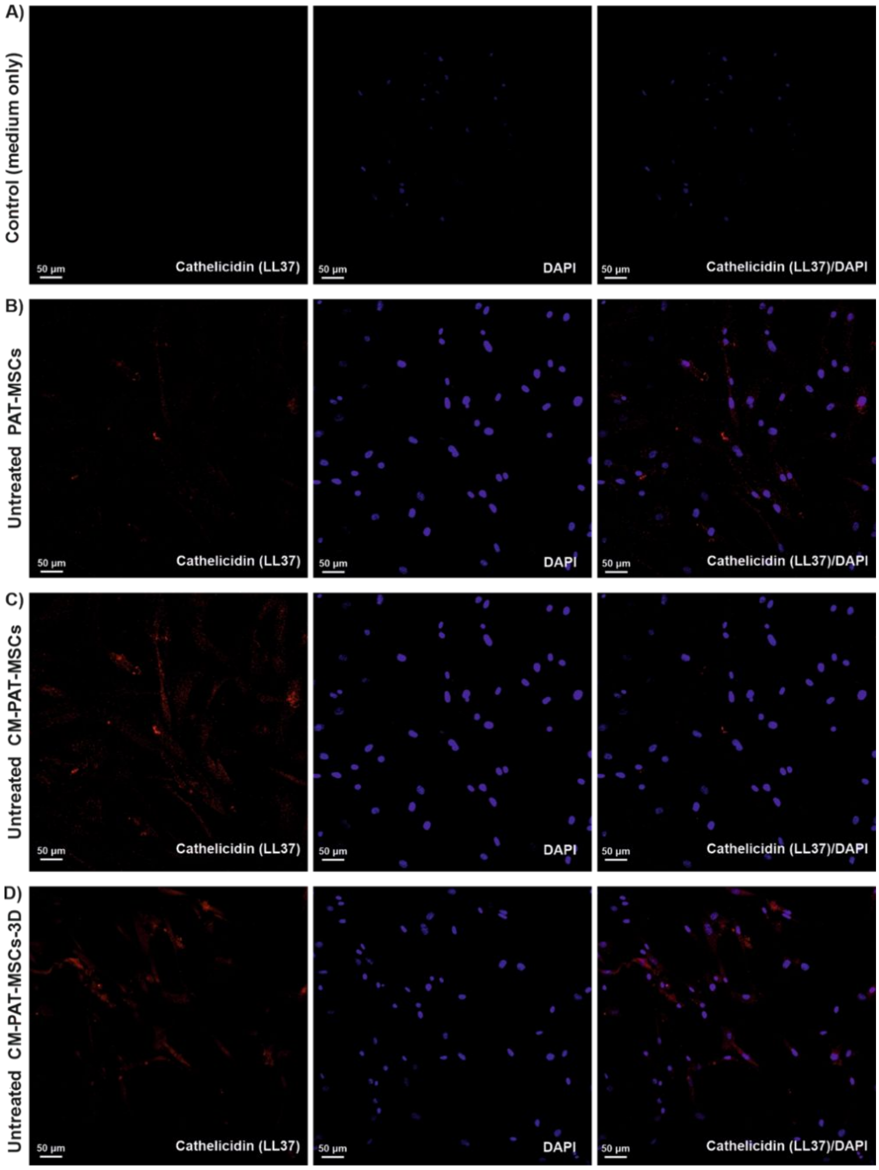

Figure S2. Representative images of untreated groups including (A) Control (medium only) without specific binding of LL-37 and nuclei staining with DAPI (blue). (B) Untreated PAT-MSCs, (C) Untreated CM-PAT-MSCs and, (D) Untreated CM-PAT-MSCs-3D. LL-37 immunostaining shown in red; nuclei counterstained with DAPI

(blue). Images were captured at a 20X magnification, scale bar = 50 μm, (n≥3 representative images for each group).
